# Reflexões pós-coloniais sobre a coleção do Centro de Arte Moderna da Fundação Calouste Gulbenkian: António Ole, Grada Kilomba, Kiluanji Kia Henda e Mónica de Miranda

**DOI:** 10.1590/S0104-59702025000100033

**Published:** 2025-08-25

**Authors:** Joana Filipa da Silva de Melo Vilela Passos

**Affiliations:** i Centro de Estudos Humanísticos/Universidade do Minho. Campus de Gualtar – Braga – Portugal Jpassos@elach.uminho.pt

**Keywords:** Arte contemporânea, Arte Africana, Decolonial, Pós-colonial, Coleção Gulbenkian, Contemporary art, African art, Decolonial, Post-colonial, Gulbenkian Collection

## Abstract

Partindo de conceitos propostos pelos estudos pós-coloniais – como a necessidade da revisão do arquivo histórico colonial, a crítica do eurocentrismo e a recuperação de memórias silenciadas –, procura-se promover uma renovação epistemológica do conhecimento ocidental, caracterizado, segundo as reconhecidas teorias de Said, Chakrabarty e Mignolo, como uma prática hegemónica, que marginaliza outras culturas e povos. Dada a atualidade e urgência dessas teorias, considera-se o reconhecimento e a valorização da arte moderna africana uma prática positiva, no sentido da renovação de ideias dominantes, transcendendo práticas eurocêntricas. Como exemplo concreto desse reconhecimento analisam-se quatro obras de artistas africanos, ou de ascendência africana, presentes na coleção da Fundação Calouste Gulbenkian, nomeadamente, António Ole, Grada Kilomba, Kiluanji Kia Henda e Mónica de Miranda.

## Abstract (en)

This article uses concepts of post-colonial studies (like the need to revise the colonial historical archive, critiques of Eurocentrism and recovery of silenced memories) to promote an epistemological renewal of Western knowledge, characterized by Said, Chakrabarty and Mignolo as a hegemonic practice that marginalizes other cultures and peoples. Given the topical and urgent nature of these theories, recognizing and valuing modern African art is a practice that can renovate dominant ideas and transcend Eurocentric practices. In a concrete example of this recognition, we analyze four works by African or African-descent artists featured in the Calouste Gulbenkian Foundation’s collection: António Ole, Grada Kilomba, Kiluanji Kia Henda and Mónica de Miranda.

No encontro científico internacional “Transmat-In2Past, Documentar coleções não europeias” (Lisboa, 22 e 23 de junho de 2023), sobre as coleções dos museus das áreas científicas da arqueologia, a etnografia e a antropologia, a minha contribuição – cuja matriz intelectual advém dos estudos literários e artísticos – dialogou com as outras perspetivas que foram apresentadas pelo questionamento dos modos de conhecimento que a própria organização de um museu impõe ao seu público, dadas as dimensões pedagógica, formativa e de lazer dessa instituição, a par do seu papel de guardiã de um dado espólio.

O enquadramento teórico que adiante se explicita fundamenta a minha abordagem a partir de teorias pós-coloniais de autores amplamente reconhecidos, como Edward Said e Dipesh Chakrabarty, com contribuições de Walter Mignolo e Grada Kilomba. Gostaria de sublinhar que os trabalhos de referência dos dois primeiros autores, embora publicados há algumas décadas, não perderam a atualidade, visto que os problemas que identificaram continuam enraizados e atuantes nas sociedades europeias e nas práticas neoliberais/neocoloniais da globalização.

Como objeto de estudo, esta pesquisa propõe uma leitura de quatro obras de arte contemporânea, unidas pela sua dimensão interventiva, ao convocar debates e problemas sociais da atualidade. António Ole, um dos artistas abordados, acredita que a sua arte é “provocadora”,^1^ e é precisamente essa qualidade que me interessa nos artistas contemporâneos selecionados para este estudo. Na condição de crítica de arte irei propor linhas de leitura para a receção desses autores, estabelecendo as relevantes conexões entre as temáticas trabalhadas pelos artistas e os discursos teóricos que as enquadram, e que, de certa forma, encaminham a receção dessas obras de arte perante um público.

O argumento desenvolvido a seguir enquadra-se no contexto dos estudos pós-coloniais – paradigma teórico sobretudo europeu e anglo-americano – que procuram repensar a história e o arquivo cultural herdados do passado colonial, corrigindo omissões, distorções e silenciamentos. Como este artigo vai ser publicado no Brasil, talvez seja relevante explicitar a distinção que faço entre os estudos pós-coloniais e o debate decolonial, teoria porventura mais corrente nesse espaço académico.

Em primeiro lugar, embora o pensamento teórico se possa aplicar a diferentes contextos, uma teoria não responde necessariamente, ou da mesma maneira, aos mesmos desafios e questões em espaços geopolíticos diferentes. Num artigo de Vera Barbosa (2023) sobre os conceitos pós-colonial e decolonial, a primeira coisa que me chama a atenção, e com a qual concordo inteiramente, é a localização geopolítica do debate decolonial no pensamento crítico “latino-americano e caribenho”, associado a formas de intervenção social e intelectual que procuram um deslocamento epistémico para além dos conceitos de modernidade e do neoliberalismo ocidental. Eu acrescentaria, a partir das minhas leituras de Mignolo, que, como alternativa à modernidade ocidental, o debate decolonial também tem afirmado a prioridade de questões ecológicas e de formas de retribuição devidas às comunidades indígenas. Ora, uma coisa é tentar recentrar o debate global a partir de uma visão que interessa sobretudo à América Latina, em função das reformas sociais que se pretendem, e da afirmação do seu contributo para o pensamento global. Outra coisa é imaginar-se (erradamente, penso eu) que esse “giro decolonial” do debate internacional cancela a validade e a urgência do debate pós-colonial, tal como foi lançado inicialmente por autores como Said e Chakrabarty. Do ponto de vista de um país europeu, com 500 anos de história colonial (como Portugal), não se pode imaginar que propostas teóricas enquadradas pelos desafios que enfrentam as Caraíbas e a América Latina dão resposta aos traumas, termos e contexto nacional(ista) da memória colonial europeia, cujo legado e consequências são ao mesmo tempo tão diferentes e tão semelhantes, de cada lado do Atlântico. Na minha opinião, a forma de demarcar com clareza o nosso caminho depende das respostas que procuramos e dos problemas que queremos resolver. E o que me preocupa nestas páginas é a receção de arte africana na Europa, o questionamento da memória colonial expondo o lado violento da expansão europeia, a mudança de paradigmas de pensamento – contrariando anteriores hierarquias e marginalizações do pensamento ocidental clássico – e, por fim, a integração da moderna arte africana nos debates da atualidade, por exemplo, em termos de ecologia e género.

Em segundo lugar, num artigo de 2016 (publicado no Brasil) procurei explicitar por que não se pode pensar que o pós-colonial é um conceito ultrapassado, a ser substituído pelas mais recentes inovações teóricas e académicas. Pensar assim é não compreender a magnitude sociológica e política do fenómeno que os estudos pós-coloniais tentam examinar. Trata-se de “entender a transição social, política e histórica vivida por diversos povos e comunidades na sequência do fim do colonialismo europeu ... que provocou, para todos os envolvidos, incluindo os colonizadores, um reajuste da conceção global do mundo” (Passos, 2016, p.107). Grande parte do processo de descolonização europeia começa depois do fim da Segunda Guerra Mundial, sobretudo nas décadas de 1950 e 1960. Como se pode imaginar que a necessária mudança de mentalidade e de ideologias dominantes, tendo em conta aquelas que moldaram a Europa colonial durante quatro séculos, poderia estar concluída algumas décadas depois? Os filhos e netos daqueles que cresceram durante a ditadura e lutaram nas chamadas “guerras coloniais” são parte significativa da sociedade portuguesa (tendo herdado várias formas de pós-memória);^2^ uma parte significativa da população portuguesa tem afiliações familiares com sujeitos que tiveram de deixar África durante o processo de descolonização (no início dos anos 1980, essas famílias vindas de África, perfaziam mais de 5% da população total portuguesa), e a sua integração com a população residente em Portugal foi um desafio social para a época, e com repercussões na atualidade. Por fim, gostaria de sublinhar que a memória histórica colonial herdada por colonizadores e colonizados é partilhada, mas não é necessariamente interpretada da mesma maneira, com atenção às mesmas prioridades. Ao nível dos desafios que a Europa enfrenta, as ideias de autores pós-coloniais de referência continuam a ser urgentes e necessárias, porque as questões que estão em discussão nesse espaço continental são-lhe específicas: ainda temos de refazer o arquivo histórico colonial europeu, a fim de incluir formas de alteridade e representação de diversidade cultural, corrigir silêncios, desconstruir modelos de conhecimento eurocêntrico e contrariar racismos ou xenofobia. Em defesa dos meus argumentos, invocaria os resultados das mais recentes eleições em vários países europeus (França, Alemanha, Itália, Holanda, e também Portugal e Espanha),^3^ que comprovaram o crescimento de uma direita xenófoba, racista, nacionalista, a funcionar em pleno dentro do paradigma ideológico da modernidade ocidental. E sublinhe-se que um dos motivos do “Brexit”, ou seja, a saída do Reino Unido da União Europeia, foi o seu desejo de autonomia para poder definir as suas próprias políticas anti-imigração. Ou seja, é inegável a urgência e centralidade dos estudos pós-coloniais, na sua forma original, mas incluindo ramificações várias, sobretudo ligadas à integração de imigrantes e diásporas que vivem dentro da Europa. Existe todo um trabalho de mudança epistémica relativa à perceção do mundo e ao lugar de diversas culturas, que ainda se tem de fazer. E a arte provoca reflexão e muda ideias feitas. Cite-se, a propósito, a entrevista que Kapil Raj concedeu a Matheus Duarte (2016), “Circulação não é fluidez”, em que o famoso historiador indiano radicado em França responde duas vezes com um rotundo “não” às perguntas sobre a abertura da Europa a outros continentes.

O que está em causa neste artigo é sublinhar o relevante contributo de artistas africanos para se transcender padrões de pensamento colonial, e, ao mesmo tempo, o importante papel da Fundação Calouste Gulbenkian, em Portugal, que, ao dar visibilidade a esses artistas, promove a procura por novas fórmulas de pensamento e novas leituras do mundo, a partir dos discursos e das ideias propostos pela arte. Uma questão anterior, aliás, e à qual a Gulbenkian tem vindo a responder ao comprar obras de artistas africanos ou de ascendência africana, tem precisamente a ver com o reconhecer-se aos artistas africanos o estatuto de autor.

Num contexto colonial, sobretudo nos séculos XVIII, XIX e princípio do XX, conceções eurocêntricas de arte e conhecimento foram impostas a outros povos, marginalizando as práticas artísticas e as heranças culturais locais como irrelevantes ou primitivas. De facto, o nativo ou o indígena produziam artefactos, mas não se concebia que pudessem produzir belas artes. A justificação para esse preconceito era sobretudo política, e partia de preconceitos racistas enraizados, tão justificáveis e credíveis quanto úteis para manter hegemonia e domínio europeus num mundo colonial. E, no entanto, representantes de todos os poderes europeus sempre se esforçaram para levar para a Europa as provas da complexidade cultural e sofisticação desses outros povos a quem negavam reconhecer humanidade. De que outra forma as muralhas da antiga cidade da Babilónia (princípio do segundo milénio a.C.), atual Iraque, foram parar a Berlim, ao Museu Pergamon? E por que o espólio do túmulo de Tutankamon está em Londres? Essa prática de desvalorizar a cultura de um povo, mas ao mesmo tempo colecionar e “recolher” os seus artefactos e espólio histórico, é prova de um discurso duplo, que desvaloriza o presente desses povos (neste caso, do Médio Oriente e Norte de África), mas não consegue esconder a admiração pelo que essas civilizações criaram.

É evidente que as iniciativas museológicas patrocinadas pelos reinos ou estados europeus procuravam trazer aos seus públicos um espólio que comprovava o domínio colonial, exibindo as particularidades dessas civilizações “outras”, que se haviam desviado do paradigma de progresso ao longo do tempo, dentro dos padrões da modernidade e da técnica, dos quais apenas a civilização ocidental auferiria. A glória de “outros” povos situa-se sempre no passado, e a sua decadência presente reafirmava, promovia, demonstrava a (suposta) necessidade da missão civilizadora do “homem branco”,^4^ materializada pelo colonialismo.

Colecionadores privados também ajudaram nesse processo de recolha dos artefactos de diversos povos situados fora da Europa. Funcionários públicos, militares, viajantes, emissários religiosos e comerciantes tornaram-se colecionadores de objetos “exóticos”, criando um mercado próprio para esses objetos, que gradualmente se tornou um circuito mais estruturado e eficiente ao longo do século XIX.

Segundo Alda Costa (2013), crítica de arte moçambicana, a receção de arte africana na Europa colonial fossilizou em torno de discursos de autenticidade, de “arte genuína”, o que queria de facto dizer “arte primitiva”, isto é, artefacto, não arte. Isto porque até meados do século XX – período de um massivo movimento de descolonização, sobretudo depois da Segunda Guerra Mundial – se considerava que as civilizações africanas não podiam criar arte moderna, urbana e original (original no sentido de produto do trabalho de um autor individual, um artista, e não o coletivo anónimo que produz o artefacto segundo o hábito ou a tradição local). Por isso, durante muito tempo, as coleções de arte africana que a Europa ainda hoje detém acabaram nos museus etnográficos, e não nos museus de belas artes, e esse preconceito é algo que as galerias de arte da atualidade têm vindo a desconstruir nas últimas décadas, com sucesso. O que está em causa, para se apreciar devidamente a arte moderna (e do passado) africana, como arte, como objeto estético, sofisticado, sublime, é precisamente renovar os modos de conhecimento, isto é, os modelos epistemológicos segundo os quais a Europa colonial se habituou a pensar ao longo de séculos.

E como os estudos pós-coloniais propõem mudar padrões de pensamento ocidental? Começo por invocar aqui as ideias de um livro que já tem algumas décadas de existência, mas que continua a ser absolutamente inspirador para o meu trabalho. Em *The world, the text and the critic*, Edward Said (1983) argumenta que a literatura (e a música) não existem no vácuo. Fazem parte do mundo, de um contexto histórico, cultural e político, posto que, como formas de arte, também servem para realizar jogos de poder, consolidando sistemas hegemónicos e ideias dominantes ou, pelo contrário, confrontando-as. O mesmo, evidentemente, se pode dizer das artes visuais, defendendo que, a par da dimensão estética, existem dimensões políticas e históricas que são convocadas pela obra de arte e que determinam a sua composição, ou criação, porque a arte existe no mundo. Interpreto, portanto, o conceito de *worldly*, de Said, como o diálogo simbiótico e visceral da arte com o mundo.

Da mesma forma que a arte é criada dentro de um contexto histórico, social e cultural, o público que a recebe também está exposto ao mesmo tipo de influências coletivas referidas, atualizando-se sob a forma de práticas de receção. Como se costuma dizer nos estudos literários, um bom leitor é um leitor que já leu muito, que tem um arquivo de referências muito vasto, e que convoca essas referências a cada vez que se sente desafiado por um texto. No caso dos autores que vou abordar, é bastante clara a intencionalidade de desafiar o público a jogar com memórias históricas do colonialismo e com preconceitos estabelecidos pela tradição de conhecimento ocidental, pondo ainda em causa silenciamentos estabelecidos e práticas epistemológicas distorcidas, que foram prevalentes durante séculos, porque o conhecimento da era colonial servia interesses políticos que determinavam o que conhecer e como conhecer. Citemos Said (1983, p.11-13):

Isto significa que a cultura (ocidental) é um sistema de discriminações e de avaliações ... perpetrado por uma classe particular que se identifica com o Estado; também significa que a cultura é um sistema de exclusões legislado a partir do topo, e executado mediante políticas que têm por função identificar anarquia, desordem, irracionalidade, inferioridade, mau gosto e imoralidade, para que sejam marginalizados em relação à cultura dominante e sejam mantidos à parte pelo Estado e as suas instituições.

...

Toda a história do pensamento da Europa do século XIX está cheia de discriminações como essas, que separam o que é adequado para nós e o que é adequado para eles.^5^

Edward Said cresceu no Cairo, no Egito, e nasceu em Jerusalém, quando Israel ainda não existia, no que era então o protetorado britânico da Palestina. Como professor célebre que exerceu toda a sua atividade profissional nos EUA, Said sabia o que era carregar dentro de si uma cultura e um lugar de pertença tornados invisíveis pelos jogos de poder do mundo. E por isso as suas palavras são tão importantes para tentar compreender os artistas que hoje em dia trabalham entre universos culturais diferentes, convocando memórias e impressões de vários mundos, que no passado tinham uma relação de poder desigual entre si.

Dipesh Chakrabarty, historiador indiano, considera que, durante o período colonial, os ingleses sempre representaram a rica, antiga e elaborada cultura indiana – que é plural e diversa, abarcando vários povos, línguas e crenças – como algo homogéneo (mais facilmente rotulável dessa forma), inadequado ou “atrasado”:

A tendência para se ler a história indiana em termos de uma falta/falha, uma ausência, ou como algo incompleto e, por conseguinte, ‘inadequado’ é óbvia nos excertos [citados]. Como conceito, representa um hábito mental antigo, que existe desde o começo da administração colonial da Índia. Os britânicos domesticaram e representaram a diversidade dos passados indianos por meio de uma narrativa homogeneizante relativa à transição do período medieval para a modernidade. Os termos mudaram ao longo do tempo. Já se chamou ao medieval ‘despótico’ e ao moderno ‘estado de direito’. ‘Feudal/capitalista’ tem sido uma variação mais recente (Chakrabarty, 2000, p.32; destaques no original).^6^

No caso português, poderemos facilmente encontrar paralelos nos textos em que se descrevem os povos e territórios colonizados por Portugal segundo a narrativa que opõe ”primitivo” a ”civilizado”, sendo que, nesse caso, a história recente é duplamente mais racista, porque vivemos grande parte do século XX sob uma ditadura cujos fundamentos ideológicos eram coloniais, conservadores, católicos, e cuja mitologia nacionalista se baseava precisamente no destino colonial português, legitimado pela tradição histórica, e por ser o precursor face a outros povos europeus. Em segundo lugar, acrescente-se a essas ideias, por longo tempo dominantes, as três frentes das guerras de independência de Angola, Guiné-Bissau e Moçambique, nos anos 1960-1970, as chamadas guerras coloniais, que duraram pelo menos 10 anos, e que ainda representam um trauma de expropriação e perda na memória coletiva portuguesa. Se considerarmos esses elementos, poderemos dizer que a efetiva descolonização de ideologias dominantes e da autoimagem nacional no caso português tem de conseguir ultrapassar não só a tradição epistemológica eurocêntrica – de que falam Said e Chakrabarty, e que equivale aos discursos ingleses sobre a Índia e o Egito –, mas também tem de ultrapassar o trauma da memória da guerra, da redução à sua dimensão europeia, e da expropriação de uma parte da sua população (aqueles que regressaram à pressa a Portugal) que bruscamente teve de se integrar na sociedade portuguesa. É pela compreensão profunda das condições históricas e sociológicas dessa tempestade perfeita que Grada Kilomba diz, tão acertadamente, que o subalterno^7^ – leia-se no âmbito do meu argumento, “o colonizado” – sempre falou, o problema é que o Ocidente, e, no caso específico, Portugal, não estava disposto a ouvir, “desqualificando” as vozes que o tentavam confrontar:

Gayatri Spivak (1995) formula a seguinte pergunta: ‘Pode o subalterno falar?’ À qual responde imediatamente ‘Não!’ É impossível à subalterna falar ou recuperar a voz, pois mesmo que ela se esforçasse com toda a sua força e violência, ainda assim a sua voz não seria ouvida ou entendida por quem detém o poder.

...

A posição de Spivak quanto à ‘subalterna silenciosa’ é, contudo, problemática se for tida como uma afirmação absoluta das relações coloniais, porque sustenta a ideia de que o sujeito negro não está apto a pôr em causa e a contrariar os discursos coloniais.

...

Não é que não tenhamos falado, mas antes que as nossas vozes – por intermédio de um sistema de racismo – têm sido sistematicamente desqualificadas como conhecimento inválido (Kilomba, 2019, p.47-51; destaques no original).

Na qualidade de investigadora portuguesa, na área dos estudos pós-coloniais, tento ouvir e compreender os discursos que os autores afro-portugueses ou portugueses de ascendência africana nos propõem hoje em dia, como forma de resistência a silenciamentos anteriormente estabelecidos pela tradição de conhecimento ocidental, herdado da era colonial. Como diz Walter Mignolo (2011), na introdução ao seu livro *The darker side of Western modernity*, a própria ideia de modernidade, algo aparentemente positivo, conotado com uma ideia de progresso e desenvolvimento, é, na verdade, um conceito colonial, que só legitima um tipo de modernidade, tal como o Ocidente o idealizou:

A tese básica ... é a seguinte: a ‘modernidade’ é uma narrativa complexa, cujo ponto de origem foi a Europa, uma narrativa que constrói a civilização ocidental ao celebrar as suas conquistas enquanto esconde, ao mesmo tempo, o seu lado mais escuro, a ‘colonialidade’. A colonialidade, em outras palavras, é constitutiva da modernidade – não há modernidade sem colonialidade. Por isso, a expressão comum e contemporânea de ‘modernidades globais’ implica ‘colonialidades globais’ no sentido exato de que a Matriz Colonial do Poder é compartilhada e disputada por muitos contendedores ...

Consequentemente, o pensamento e a ação decoloniais surgiram e se desdobraram, do século XVI em diante, como respostas às inclinações opressivas e imperiais dos ideais europeus modernos projetados para o mundo não europeu, onde são acionados (Mignolo, 2017, p.1-2).

Onde Mignolo vai mais além do que outros autores referidos – que já reconhecem e demonstram que o discurso colonial era tendencioso, manipulador e racista – é no facto de propor uma prática de roturas, de quebras e recusas – que é exatamente o modo decolonial –, promovendo a consolidação da validade internacional de modelos epistémicos outros, sobretudo provenientes das culturas indígenas, da ecologia ou, acrescentaria eu, da comunidade afro-brasileira.

No que nos diz respeito a este artigo e tendo em conta a tradição do mercado de arte europeu, o que está em causa é o reconhecimento internacional da qualidade da arte moderna africana, algo impensável até há algumas décadas. Reconhecer esses autores é um gesto que transcende o paradigma de pensamento eurocêntrico e colonial. Assim, a coleção de autores africanos dentro do Centro de Arte Moderna da Fundação Calouste Gulbenkian é um passo em frente, que deve ser estudado e celebrado como exemplar.

Outra inovação interessante, a partir do outro lado do Atlântico, é o caso da Bienal de São Paulo de 2023. Que impacto terá perante o público português (ou outros públicos europeus) uma exposição que se apresenta como “Uma bienal para tentar desmontar o tempo colonial, progressivo e ocidental”? Esse é o título de uma reportagem do *Público* (um dos jornais de maior circulação em Portugal), de 5 de junho de 2023, que também apresenta o quarteto de curadores responsável por essa bienal, que se pretende, segundo o referido artigo, “disruptiva”. Três dos curadores definem-se como não brancos: Diane Lima, Grada Kilomba e Hélio Menezes, e só o espanhol Manuel Boria-Villel se qualifica como branco. Também existe paridade de género: dois homens e duas mulheres, e, em lugar de um curador, temos um coletivo, que resolve tudo em conjunto. A maior parte dos artistas que expõem também não é de “estrelas internacionais do mundo da arte contemporânea” (Salema, 2023, p.28). Claramente é uma bienal concebida de uma forma diferente, eu diria exploratória, no sentido idealizado por Mignolo, de tentar pensar paradigmas inovadores, para transcender heranças epistemológicas do mundo colonial e eurocêntrico. Said e Chakrabarty certamente concordariam.

## A Fundação Calouste Gulbenkian e o Centro de Arte Moderna

Como referência credível e muito respeitável do que é a cultura e a arte, a Gulbenkian tem desenvolvido um trabalho coerente no sentido de transcender a tradição de pensamento ocidental, de raiz colonial.

A fundação foi constituída em 1956, com um fundo legado a Portugal, por testamento, pelo cidadão arménio Calouste Sarkis Gulbenkian. Desde então, a fundação tem por missão “melhorar a qualidade de vida das pessoas através da arte, da beneficência, da ciência e da educação”,^8^ o que tem efetivamente feito, com grande reconhecimento e prestígio internacionais. A Fundação Calouste Gulbenkian, com sede em Lisboa, tem filiais em Paris e Londres, e programas internacionais de apoio aos países africanos de língua portuguesa e a Timor-Leste, bem como às comunidades arménias. Trata-se de instituição progressista, referência de cultura e qualidade, e um dos principais patronos da cultura e das artes em Portugal. De entre as suas várias valências, conta-se o Centro de Arte Moderna (CAM), detentor de uma das mais importantes coleções de arte em Portugal, com projeção internacional.

Inaugurado em 1983, o CAM ocupa desde 2024 seu novo edifício, projeto do arquiteto japonês Kengo Kuma. O sistema rotativo de exposições permite ao CAM mostrar diferentes coleções dentro do seu vasto espólio, que inclui uma ampla e representativa coleção de arte moderna portuguesa, de cuja divulgação, aliás, o centro tornou-se um espaço de referência. A aquisição das obras que compõem essa coleção segue a lógica do mercado internacional, mas a sensibilidade especial da Gulbenkian para escolher artistas e obras de arte responde também aos objetivos da própria fundação, criada para promover o bem comum e o conhecimento, contribuindo ativamente para tentar tornar o mundo um local melhor.

Citemos, a título de exemplo, a exposição Europa Oxalá (4 de março a 22 de agosto de 2022) que integrou 60 obras de arte, além de livros, catálogos e revistas de arte (englobando a crítica, a teoria e a história de arte) focados em artistas nascidos em África ou da diáspora africana, ou nascidos na Europa, mas refletindo sobre memórias coloniais. Também o ciclo de conferências Próximo Futuro, em 2015, coordenado por António Pinto Ribeiro, marcou um momento inédito nos debates da sociedade portuguesa, pela dimensão e pela multiplicidade de iniciativas (exposições, ciclos de cinema, espetáculos) que divulgaram perante o público português representações, discursos e ideias de autores árabes, africanos ou da América Latina. Inclusivamente, Walter Mignolo foi um dos oradores convidados.

No caso dos artistas de origem africana representados no CAM, decidi selecionar quatro nomes emblemáticos, reconhecidos e com carreira internacional: António Ole, Grada Kilomba, Kiluanji Kia Henda e Mónica de Miranda. De cada um desses autores, será analisada uma obra, sinalizando as interrogações e os desafios que eles nos lançam. Ao mesmo tempo, sublinha-se que as obras selecionadas vão ao encontro das denúncias e preocupações expressas pelos teóricos pós-coloniais já aqui citados, juntando-se neste artigo os discursos teóricos revisitados e a obra criativa dos autores escolhidos – como as duas faces do mesmo gesto provocador, da mesma vontade de interpelar o público.

## António Ole

Artista angolano, nascido em 1951, com carreira internacional consolidada, António Ole tem tido sua carreira acompanhada há vários anos pela Gulbenkian, que não só adquiriu várias obras suas para a coleção da fundação como organizou a exposição retrospetiva “António Ole. Luanda, Los Angeles, Lisboa”*.* Nessa exposição, estiveram em destaque os enormes murais que invocam as construções improvisadas dos musseques (as favelas de Angola), a partir de desperdícios e materiais diversos, de origem díspar e desorganizada. Assim sublinhava Ole a vivência improvisada e frágil dos que (sobre)vivem a partir de restos e sobras, materiais desvalorizados por quem tem acesso a uma vida melhor. Com curadoria de Isabel Carlos e Rita Fabiana, essa grande exposição, patente entre 16 de setembro de 2016 e 9 de janeiro de 2017, deu origem a um catálogo, que inclui a lista das obras em exposição, uma entrevista com António Ole, uma biografia (afinal, Ole tem atrás de si uma carreira de 50 anos) e um texto crítico sobre os temas da sua obra, nomeadamente, o mar, as cidades e a memória. Nadine Siegert, que contribuiu com um ensaio para o catálogo acima citado, debruçou-se sobre o trabalho de António Ole e Kiluanji Kia Henda num artigo (Siegert, 2017) em que argumenta ser a obra desses artistas interventiva e comprometida com uma reflexão sobre a memória colonial, vista a partir de uma perspetiva crítica que tenta articular contramemórias e contradiscursos, isto é, criar alternativas a ideias feitas e discursos estabelecidos.

De um ponto de vista europeu, no qual se insere Portugal e de onde partem as políticas de aquisição de arte da Gulbenkian, a receção de artistas como António Ole é absolutamente necessária para, à luz das teorias pós-coloniais, se renovar o conhecimento herdado de modelos coloniais, corrigindo anteriores distorções, moldadas a partir de uma visão eurocêntrica, hegemónica e racista, que marcou o pensamento ocidental durante séculos. No caso deste artigo, interessa-nos precisamente a vertente da obra de António Ole que dialoga com a memória histórica.

Por outro lado, é verdade que a obra de Ole é bastante rica, cobre muitas temáticas, e pode ser abordada de outros vários ângulos. Por exemplo, a crítica de arte Ana Balona de Oliveira (2021) publicou um artigo sobre a vertente ecológica da obra de António Ole, realçando a sua atenção a materiais e texturas naturais, por meio de fotografias da natureza de Angola. Não será esse, porém, o nosso foco de atenção.

Neste segmento do artigo queria ater-me a um mural de António Ole adquirido pela Gulbenkian^9^ em 2016, composto por oito composições quase idênticas, dispostas em fila, mais uma composição em destaque, encimada por um pedaço de madeira (Ole, 1996-2001). Essa instalação incluía uma projeção (vídeo) que não vamos abordar, e é composta por vários materiais, nomeadamente, tela, ossos, lanternas e pratos de esmalte, entre outros objetos. O mural está datado de 2001, e o seu título é *Hidden pages, stolen bodies* (Páginas escondidas, corpos roubados). Na folha de sala *online* da Gulbenkian pode ler-se que essa composição aborda “um passado mais sombrio: conta a história negligenciada de vidas marcadas pelo trabalho forçado sob o domínio colonial no final do século XX” (Lopes, 28 mar. 2022). Ora, eu acho que podemos ir muito mais longe na interpretação dessa composição, e a minha chave para interpretar esse trabalho é a tábua de madeira, que parece ter sido ignorada na descrição citada. Essa tábua é um destroço, e a parte invertida tem o recorte para se encaixar o remo. Trata-se do destroço de um bote e, associado às fotografias dos corpos negros, debilitados, magros, evoca imediatamente a história da escravatura e o transporte de escravos. São páginas escondidas da história, pois nenhum país está confortável com o seu passado escravocrata, e são corpos roubados, às suas famílias, às suas comunidades, ao seu país. A ideia de que a história dos escravos é uma história anónima está plasmada na moldura sem retrato, e na reprodução do mesmo corpo sem face nos vários quadros do mural. Não sabemos quem eram esses escravos, mas sabemos que eram muitos, e que a sua história se repetia. Por fim, destacaria a lanterna no último quadro à esquerda. Pode ser a lanterna de um barco, e os pratos e chávenas de zinco tanto evocam a alimentação dos escravos como das vítimas de trabalho forçado. Seja na minha interpretação, seja na da página da Gulbenkian, em ambos os casos, António Ole recupera uma história silenciada, as suas páginas perdidas, sinalizando a necessidade de se fazer o arquivo desse passado e dos corpos roubados, mal alimentados, cujas identidades se perderam. Sua obra responde às preocupações dos já mencionados Said, Chakrabarty, Mignolo, Kilomba, pois conta uma versão de acontecimentos históricos do ponto de vista do subalterno, corrigindo o arquivo de conhecimento histórico de forma a integrar o lado da história que foi silenciado no conhecimento público e corrente do que foi o colonialismo. Contraria-se assim o poder do conhecimento ocidental estabelecido, revelando o preço humano da modernidade/colonialidade, que se revela obscura, cruel e letal, como diz Mignolo.

## Grada Kilomba

Na prestigiada Pinacoteca de São Paulo, Grada Kilomba teve uma exposição intitulada Desobediências Poéticas, que esteve aberta ao público de 6 de julho a 30 de setembro de 2019. O catálogo dessa exposição tem, na capa, parte da fotografia que é analisada neste artigo (Grada Kilomba..., 2019). Sublinho essa coincidência na escolha do mesmo objeto estético porque, de certa forma, valoriza a minha seleção de objeto de estudo, uma vez que a própria artista terá acompanhado a elaboração do catálogo, e escolheu destacar essa fotografia.

Um dos principais focos da obra de Grada Kilomba, quer na componente artística, quer na sua dimensão teórica, tem a ver com o lugar dos “corpos negros” e da “mulher negra” na sociedade ocidental, consolidada segundo o ponto de vista determinado pelos valores e visões da “branquitude”. A propósito, recordaria também o impacto do seu livro *Memórias da plantação, episódios de racismo quotidiano* (Kilomba, 2019),^10^ que foi a sua tese de doutoramento na Alemanha. Em suas páginas, Kilomba procura expor uma micro-história de repetida opressão e violência sobre os corpos negros, em pequenos gestos do quotidiano. Alternativamente, desenvolve novas formas de representar, isto é, de dar a ver, os corpos racializados pelos discursos da branquitude como corpos “normais”, despidos de preconceitos, isto é, das ideias feitas, que os têm predefinido e predeterminado.

Em recente artigo na *Revista de Estudos Feministas*, Guilherme Marcondes e Roberto Marques (2022, p.11) abordam a confluência entre o trabalho de Grada Kilomba e outras autoras ou artistas que se debruçam sobre a

formação da nação, questões raciais, de gênero e classes sociais, ... [pois] é preciso demarcar de onde se fala. Ou melhor, é preciso compreender negrodescendentes como ‘sujeitos’, e não somente ‘objetos’.

...

Efetivamente, cremos ser possível dizer que tanto Kilomba quanto Bicudo contribuem para o processo de descolonização da sociedade ocidental. Suas análises são propositivas. ... Ambas as autoras revelam a estrutura racista que fundamenta as sociedades que analisam e, assim, indicam outras possibilidades de ação. Deste modo, seus trabalhos podem ser até mesmo compreendidos como reações às ações dos opressores, colocando-os desnudos, buscando explicitar traumas gerados em negrodescendentes e indicando outros caminhos societários.

Esse comentário, que me é muito útil para apresentar aqui o trabalho de Kilomba, é igualmente esclarecedor para se compreender a forma como distingo o decolonial do pós-colonial como modelos de pensamento crítico a partir do lugar “de onde se fala”. O decolonial foca-se no poder transformativo (propositivo) de novas ideias e aponta as feridas, a agressão infligida pelo conhecimento ocidental. Mas, como o pós-colonial demonstra, não se pode simplesmente propor uma rotura radical com modelos de pensamento colonial pela simples razão de que o Ocidente, sobretudo a Europa, não vai viver o futuro a partir de um momento de amnésia coletiva, que não aconteceu. Subsistem arreigados sentimentos nacionalistas nos países europeus, e subsiste uma transversal visão hegemónica da história europeia que é preciso desconstruir. Implicitamente, o pós-colonial está a criar o espaço para se receberem novas ideias e novas visões, mas as novas ideias serão simplesmente ignoradas se não voltarmos o conhecimento europeu contra si próprio (o que o pós-colonial tenta fazer). Veja-se o caso do argumento deste artigo: só foi possível estudar os autores aqui abordados a partir do momento que instituições como a Gulbenkian contribuíram para que se reconhecesse aos artistas e autores africanos o seu estatuto de autor. Se não se questionasse a tradição da crítica de arte, que simplesmente os ignoraria, não se criaria o espaço para ouvir e compreender as críticas feitas ao conhecimento ocidental. Logo, sem os estudos pós-coloniais, a receção de uma visão decolonial do mundo pode ser recebida com desatenção ou até indiferença. Os modelos de conhecimento que tentam reduzir desigualdades devem, portanto, ser entendidos como complementares, e não como rivais. Mesmo dentro da complementaridade, no entanto, será sempre necessário compreender o que cada um deles está a fazer, atuando num dado contexto geopolítico, que é o seu.

A fotografia de que nos vamos ocupar faz parte da trilogia de Grada Kilomba (originalmente vídeo e *performance* teatral) *A world of illusions*. Essa *still* foi adquirida pelo CAM em 2021. De acordo com a folha de sala disponibilizada pela Gulbenkian, as fotografias, além de serem objetos estéticos em si, também remetem para as *performances* teatrais com atores e atrizes, nas quais Kilomba reescreveu mitos clássicos. Não me vou debruçar sobre as *performances*, que foram acontecimentos dinâmicos e momentâneos. O que a Gulbenkian guarda no seu arquivo são fotografias, que são composições autónomas. Vou analisar aqui uma delas (Kilomba, 2018), em que se pode ver a autora a encarnar a esfinge que recebe Édipo, quando este chega à cidade de Tebas.

Nessa fotografia, imagem fixa que isola um momento da narrativa, o cenário minimalista baseia-se num forte contraste entre preto e branco, o que é importante para sublinhar a presença dos corpos negros num imaginário ao qual não estão tradicionalmente associados, que é o da cultura greco-latina, referência fundadora para o mundo ocidental. Afinal, nessa imagem subversiva, quem personifica os mitos que nos transmitiram um saber eterno é um corpo africano, o que joga ironicamente com expectativas e referências pré-estabelecidas na cultura europeia, e subverte a tal tradição do conhecimento de que falei. E, de facto, a própria ideia de se reescrever a mitologia clássica já é um exemplo de desconstrução da tradição do pensamento clássico ocidental.

É de destacar que a imagem composta é muito clara, facilmente reconhecível por quem tiver um mínimo de referências da cultura clássica, logo, eficaz no objetivo de permitir ao público identificar o mito que se invoca, e qual o imaginário que está em causa. Representa-se um momento da tragédia clássica *Édipo Rei*, de Sófocles. Nesse quadro, Édipo chega à cidade de Tebas sem saber a sua verdadeira identidade (é o filho do rei, e havia sido banido na infância). A entrada de Tebas é guardada pela esfinge, que lhe vai lançar um desafio: se Édipo não responder acertadamente ao enigma, ela mata-o.

Na fotografia em causa, momento inicial do confronto, é a esfinge que detém o conhecimento e o poder, pois é ela que testa os viajantes com o seu enigma. Sendo assim, Grada Kilomba isola um momento que subverte toda a história dominante: antes de ser derrotada, é a esfinge/mulher negra quem detém o poder e a sabedoria, enquanto um Édipo atento tenta estar à altura do desafio. Édipo é o humano perdido, que não se conhece a si próprio, a esfinge é um ser sobrenatural, que pertence a um plano superior.

Para além da subversão de hierarquias de raça e de género propostas por essa imagem, queria também apontar que o mundo clássico – aquele que o Ocidente toma como referência próxima em termos de civilização, e que representa como a “sua” antiguidade – é, na verdade, um produto do mundo mediterrânico, e o Mediterrâneo sempre foi navegado; logo, as pessoas das duas costas, Sul da Europa e Norte de África, sempre circularam, se misturaram e influenciaram mutuamente. A prova disso é que a esfinge, figura mítica com corpo de leão e cabeça de mulher (em algumas versões, alada), tanto existe na mitologia grega como no mundo egípcio. Por que se toma então a cultura clássica greco-latina por um conjunto de referências culturais exclusivamente europeias e brancas? As fronteiras culturais são afinal bem mais porosas e indefiníveis do que fronteiras políticas, e não são rígidas. Os mitos e as ideias podem viajar e influenciar vários povos, recebendo em troca influências várias. Por isso mesmo, Grada Kilomba (1968- ), portuguesa de ascendência angolana e são-tomense (acho importante referir essas identidades compósitas, de múltipla pertença), revisita três mitos clássicos, Narciso, Édipo e Antígona, reinterpretando-os, e reinterpretar é também reclamar um direito de posse, um direito a partilhar significados, sinalizando a natureza miscigenada das heranças culturais do mundo, sobretudo num contexto de tão intensa navegação, como o era o Mediterrâneo, mesmo nos séculos antes de Cristo.

## Kiluanji Kia Henda

Kiluanji Kia Henda (1979- ), que já teve exposições individuais em vários países, como Bélgica, África do Sul, EUA, Itália e Portugal (bem como inúmeras participações em exposições coletivas), foi selecionado em 2020, por votação da Associação de Afrodescendentes Portugueses, para fazer, em Lisboa, o Memorial de Homenagem às Pessoas Escravizadas (em local ainda em discussão até à escrita deste texto). Kia Henda participou da Bienal de Veneza (20 de abril a 24 de novembro de 2024) em representação de Angola, com conjuntos de fotografias sobre gradeamentos, um deles relativo a gradeamentos de proteção em habitações privadas, e outro com paisagens da Sardenha, vistas através de grades. Segundo a curadora Tarini Malik, essas grades fotografadas por Kia Henda recordam-nos as barreiras “físicas e legais, com as quais o continente europeu se equipou para responder à chegada de ondas de emigrantes nos últimos anos” (Kiluanji..., 2024). Mais uma vez se enquadra a receção de obras de arte num contexto situado, europeu, que responde a problemas regionais específicos, como tenho defendido ao longo deste artigo.

Neste artigo abordo o trabalho *Natureza quase morta* (Henda, 2009), que foi adquirido pela Gulbenkian em 2018. Escolhi esse trabalho porque a preocupação com a ecologia é sem sombra de dúvidas um dos mais pertinentes debates da atualidade, e achei que seria importante sublinhar que a criatividade contemporânea de autores africanos não se reduz a pensar a sua relação com o Ocidente e a história colonial, embora essas sejam, sem dúvida, temáticas centrais na produção de autores conscientes dos desafios e problemas atuais, sobre os quais também temos de refletir. O que nos prende o olhar nessa fotografia é a sensação de ameaça pendente. Aquela máquina vai destruir o espaço onde aquela palmeira sobrevivia. Os postes sem rede demarcam um espaço que será fechado, circunscrito, e aquelas máquinas imensas e aqueles postes vieram mudar o cenário natural. A palmeira, isolada e muito mais pequena do que as máquinas, é a vítima que se adivinha, num cenário que será desertificado, e que se tornará um espaço sem vida pela interferência de mão humana. Kiluanji Kia Henda faz aqui o cruzamento entre questões ambientais e a destruição da natureza pela tecnologia, para servir a grandes projetos empresariais.

Sua obra dialoga sobretudo com os ensaios de Walter Mignolo sobre a visão da natureza como recurso, ou como matéria inerte para ser usada em função do lucro capitalista. Trata-se de uma visão moderna e ocidental, totalmente oposta à visão integrada e mística que os povos indígenas têm da sua relação com a natureza. De um ponto de vista pós-colonial, essa fotografia pode ser lida como uma acusação, responsabilizando aqueles que destroem/têm vindo a destruir a natureza em função do lucro, visto que o projeto colonial foi moldado por objetivos de acumulação de riqueza a partir de exploração das matérias-primas das colónias.

## Mónica de Miranda

Mónica de Miranda representou Portugal na Bienal de Veneza de 2024, juntamente com Sónia Vaz Borges e Vânia Gala. É uma artista luso-angolana, fundadora do Hangar, centro de arte e investigação em Lisboa. Como académica, Miranda é a coordenadora do projeto de investigação intitulado “Pós-arquivo: política da memória, lugar e identidade”, acolhido pelo Centro de Estudos Comparatistas da Universidade de Lisboa.

Aqui abordo uma *still* do filme *Path to the Stars*, de 2022, que faz parte de um filme/vídeo dirigido por Mónica de Miranda.

Apesar da paz dessa imagem, que até nos sugere relaxamento, é impossível não ver essa mulher como um soldado, e essa é, na minha opinião, a questão central que essa imagem convoca. Essa não é uma fotografia de guerra nem de ação. Por isso é tão importante a linguagem corporal da figura humana retratada. Essa mulher em uniforme, integrada nessa paisagem isolada, de mato, que é onde estava a guerrilha, evoca o papel fundamental que as mulheres tiveram em vários movimentos de libertação. Muitas vezes, o regime pós-independência esqueceu as mulheres ao construir a sua própria narrativa da luta pela autonomia. Reconhecer o papel histórico, e heroico, dessas mulheres é algo que já começou a acontecer, mas que ainda tem de ser incentivado. Aliás, a natureza não panfletária do trabalho de Mónica de Miranda pode medir-se pela languidez tranquila dessa personagem. Já não precisamos de mitos militares nem de *slogans* que encorajam a ação e chamam para a luta. Mas, em tempo de paz, não se pode esquecer que as mulheres também envergaram uniformes, quando foi necessário. Esse trabalho de Mónica de Miranda (2002) cruza os debates pós-coloniais e as questões de memória histórica com questões de género e o silenciamento do contributo das mulheres para várias causas públicas e históricas. Ao refazer-se o arquivo histórico, ao corrigir-se o arquivo de conhecimento ocidental e global, bem como enraizados hábitos epistemológicos sexistas, não se pode esquecer as reivindicações dos estudos de género, pois é fundamental, neste processo reparador que implica repensar ideias feitas e as práticas que essas ideias legitimavam, ter em conta o contributo e o valor das mulheres, numa lógica de igualdade de géneros, que se entrelaça com a igualdade de raças e de civilizações. Ao mesmo tempo, se enquadrarmos essa fotografia no *trailer* que apresenta o filme, é inegável o protagonismo das mulheres em imagens que evocam memórias fúnebres do pós-guerra, como a imagem de um soldado morto, ou a presença silenciosa de figuras estáticas, possíveis representações de entes sobrenaturais/desencarnados.

## Considerações finais

Por meio desses quatro exemplos, espero ter demonstrado como as diferentes obras abordadas atualizam (e dialogam com) discursos teóricos que são referências para se pensar o papel da arte hoje em dia. Espero ter também demonstrado como a arte de facto não existe no vácuo, sendo inspirada pelo mundo, pela história e por jogos de poder. No caso específico do pensamento pós-colonial, uma matriz fundamental para interpretar as obras selecionadas é a relação da arte contemporânea com a memória histórica, que por intermédio de uma arte interventiva e comprometida se convoca, analisa e desconstrói.

Paralelamente, ao trazer aqui a coleção de artistas africanos ou de ascendência africana do Centro de Arte Moderna da Gulbenkian também se demonstra o impacto e o reconhecimento que esses artistas têm, e reafirma-se a sua centralidade nos circuitos internacionais de arte. Por fim, ao relacionar o discurso de vários pensadores fundamentais nos debates teóricos da contemporaneidade com as práticas desses quatro artistas, demonstra-se como essas obras de arte convocam temas e debates tão urgentes como relevantes para se conceber o futuro em moldes diferentes dos padrões de pensamento do passado, que se pautaram pela gestão e preservação de várias formas de injustiça, marginalização e violência.

**Figura 1:**
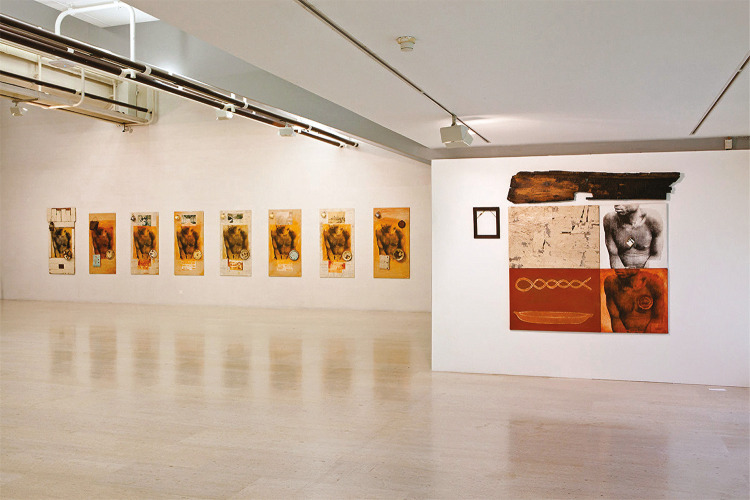
: Mural de António Ole, 2001, *Hidden pages, stolen bodies;* foto de Carlos Fernando Esteves Azevedo (Centro de Arte Moderna Gulbenkian, Lisboa)

**Figura 2:**
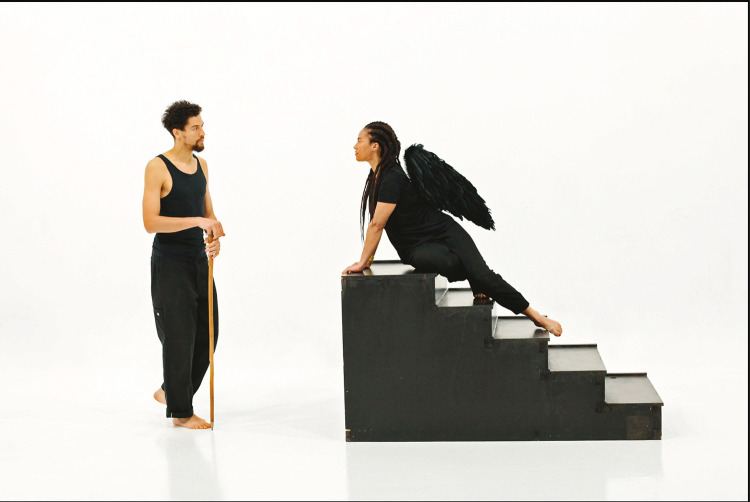
: *Still* de vídeo de Grada Kilomba, 2021, *Illusions vol. II, Oedipus*, da trilogia *A world of illusions*; foto de Carlos Fernando Esteves Azevedo (Centro de Arte Moderna Gulbenkian, Lisboa)

**Figura 3:**
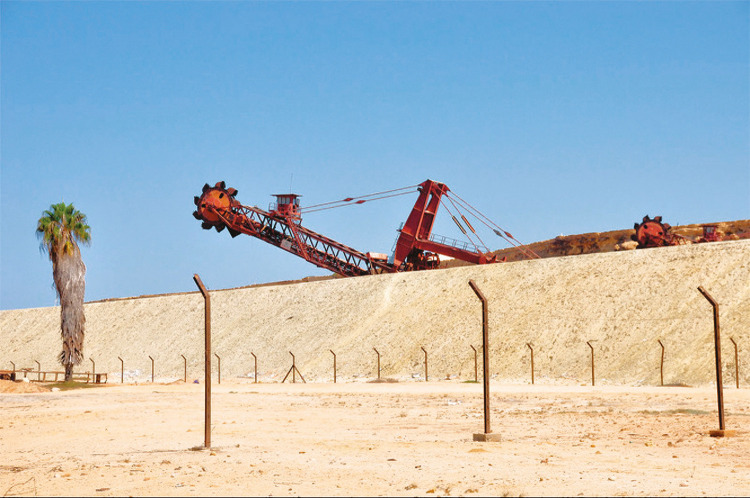
: Kiluanji Kia Henda, 2009, *Natureza quase morta* (Centro de Arte Moderna Gulbenkian, Lisboa)

**Figura 4:**
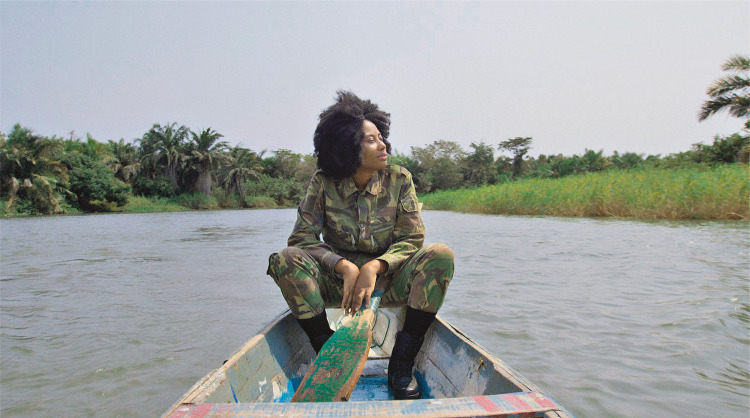
: Mónica de Miranda, 2022, *still* do vídeo *Path to the stars* (Centro de Arte Moderna Gulbenkian, Lisboa)

## Data Availability

Não estão em repositório.

## NOTAS

1 “Acredito que a minha arte possa ajudar as pessoas a mudar, porque é sempre provocadora” – frase de António Ole, citada em Exposição... (2009).

2 Guerras de libertação em Angola (1961-1974), na Guiné-Bissau (1963-1974) e em Moçambique (1964-1974). Em Portugal, essas guerras de libertação também se designam por guerras coloniais.

3 No caso concreto de Portugal refiro-me ao aparecimento de um partido de extrema-direita, o Chega, que neste momento (2024) tem nada menos do que 50 deputados, do total de 230, na Assembleia da República Portuguesa. Nas eleições para colocar deputados no parlamento europeu, o VOX, partido espanhol de extrema-direita, foi o terceiro partido mais votado, tendo recebido 9,6% dos votos. O partido que elegeu mais deputados também é de direita, o Partido Popular, com 34% dos votos e 22 deputados eleitos; e surgiu outro partido de extrema-direita, nacionalista e xenófobo, AaF, que ainda conseguiu 4,6% dos votos.

4 Veja-se, a propósito, o famoso e emblemático poema “The white man’s burden” (O fardo do homem branco), publicado pela primeira vez em 1899 por Rudyard Kipling, prémio Nobel da literatura em 1907, e que representa a matriz de uma visão colonial (e racista) do mundo.

5 Nessa e nas demais citações de textos em idiomas estrangeiros, a tradução é livre. No original: “This means (Western) culture is a system of discriminations and evaluations … for a particular class in the State able to identify with it; and it also means that culture is a system of exclusions legislated from above but enacted through its polity, by which such things as anarchy, disorder, irrationality, inferiority, bad taste, and immorality are identified, then deposited outside the culture and kept there by the power of the State and its institutions. … The entire history of nineteen-century European thought is filled with such discriminations as these, made between what is fitting for us and what is fitting for them.”

6 No original: “The tendency to read Indian history in terms of a lack, an absence, or an incompleteness that translates into ‘inadequacy’ is obvious in these excerpts. As a trope it is ancient, going back to the beginnings of colonial rule in India. The British conquered and represented the diversity of Indian pasts through a homogenizing narrative of transition from a medieval period to modernity. The terms have changed with time. The medieval was once called ‘despotic’ and the modern ‘the rule of law.’ ‘Feudal/capitalist’ has been a later variant.

7 Ao utilizar esse termo, Grada Kilomba está a evocar um ensaio fundamental nos estudos pós-coloniais, o famoso ”Can the subaltern speak?”, da pensadora indiana Gayatri Spivak (1995), com o qual dialoga no seu próprio texto.

8 Informações disponíveis em: https://gulbenkian.pt/.

9 As práticas de aquisição de obras da arte da Fundação Calouste Gulbenkian regem-se pela lógica do mercado atual, legal, de obras de arte. Todos os artistas abordados são contemporâneos e são representados por galerias de arte.

10 A edição original é de 2008; em 2019 foi publicada a primeira edição em português.
